# A selective genotyping approach identifies QTL in a simulated population

**DOI:** 10.1186/1753-6561-8-S5-S5

**Published:** 2014-10-07

**Authors:** Bianca Moioli, Francesco Napolitano, Gennaro Catillo

**Affiliations:** 1 Consiglio per la Ricerca e la sperimentazione in Agricoltura, via Salaria 31, 00015 Monterotondo, Italy

## Abstract

**Background:**

Identification of QTLs for important phenotypic traits, through the use of medium-density genome-wide SNP panels, is one of the most challenging areas in animal genetics, for preventing the time-consuming direct sequencing of putative candidate genes, when searching for the mutations that affect the trait. Appropriate statistical analyses allow the identification of genomic regions associated with the investigated trait in the genotyped population.

**Methods:**

The selective genotyping technique was applied to 1000 genotyped animals with known phenotype. Sliding windows composed of five consecutive SNPs were created for each chromosome; we assumed that the QTLs were encoded by the windows showing the highest difference in the frequency of the same alleles between the most divergent productive groups (the two tails of the distribution).

**Results:**

Ten windows affected at least one trait. For five of these windows, the highest and significant effect was given by one only SNP, which could therefore be taken as the QTL itself.

**Conclusions:**

In this study we proposed a simple method to identify genomic regions associated to the phenotype under study. The identification of the DNA region is the first step to search for the mutation which is really responsible for the trait variability, through the direct sequencing of the genome regions that encode the QTL.

## Background

The recent availability of genome-wide SNP panels, which offered the opportunity to evaluate the variation in SNP allele frequencies between populations, allowed the successful finding of genomic regions subject to positive selection in human and cattle [1-5]. For the identification of selection sweeps for milk traits, efficient application of the selective genotyping strategy for QTL mapping has been reported in dairy cattle [6], swine [7] and sheep [8]. In these cases, the extreme divergent individuals for a trait (the two tails of the distribution) are chosen and genotyped. Boligon et al. [9] compared selective genotyping strategies for prediction of breeding values in a population undergoing selection, and concluded that animals with extreme yield deviation values in a reference population are the most informative when training genomic selection models. Using the selective genotyping approach, Moioli et al. [8] identified two novel non-synonymous mutations associated with milk yield in sheep, and demonstrated their effect also in independent populations.

In the present study, we hypothesized that selection sweeps, detected in a simulated population, were useful to map QTLs for the trait under selection in the whole population.

## Materials and methods

### Dataset

Three milk production traits were simulated in a population of 3,000 females, included in a data set of 4,100 individuals of 4 different generations (G0 to G4) having known pedigree. Females and parental genotypes at 10,000 SNPs equally distributed on 5 chromosomes were available. A detailed description of the population is reported by Usai et al. [10].

### Statistical analysis

The selective genotyping technique was simulated on the females of generation 3 (1000 females), assuming that they were those who had better profited of the selection. Their production was reported on table 1. Allele frequencies at each SNP of each chromosome were calculated separately for the group the production of which was <-1 st dev for each trait, and the group the production of which was >1 st dev for each trait. The number of the animals of each group was also reported in table 1. The QTLs so hypothesized might be affected by the number of individuals included in the production tails, this depending on the additive-relationship between them, which might not represent the average relationship of the whole population. Habier et al. [11], in the context of predicting genomic breeding values (GEBV), advised that additive-genetic relationships between the training individuals and a selection candidate, captured by SNPs, affects the GEBV accuracy of that candidate. Therefore, in the present study, coefficient of relationship between the individuals of each tail portion, as well as the whole population were calculated as in Wright [12] using Proc Inbreeding in SAS [13].

**Table 1:** Statistical parameters relevant to the analyzed traits in the female population of generation 3

Variable	N	mean	st dev	min	max	N< -1 st.dev	N> 1 st.dev
Trait1	1000	-6.42	171.32	-526.43	483.17	173	150
Trait2	1000	-0.170	9.60	-32.23	25.51	165	167
Trait3	1000	0.000396	0.02388	-0.089	0.085	156	153

The QTL effect was subsequently estimated with the use of sliding windows, composed of five consecutive SNPs and calculated for each of the five chromosomes. The number of markers in each window was established based on the consideration that the SNP density of the simulated population of the present study was similar to the average SNP density of the cattle panel used by Stella et al. [2]. These authors suggested that sliding windows of 5, 9, and 19 SNPs respectively give similar results when searching for selective sweeps in cattle.

For each window, the sum of the differences (in absolute value) of the allele frequencies, at each SNP, between the two productive groups, was calculated; the sliding windows were then ranked, according to this parameter, within each chromosome. We arbitrarily hypothesized that the potential QTL, for the considered trait, was located in the top ranking window. Because the selective genotyping was performed separately for the three traits, the potential QTLs could be located in different windows; for this reason, more than one window in the same chromosome were considered in the subsequent analyses.

The top ranking sliding windows, encoding the hypothesized QTL, as well as the potentially affected traits, are reported in table 2.

**Table 2:** Top ranking sliding windows based on the highest difference in allelic frequencies between the two productive groups, separately for each trait

chr	Starting position	End position	markers	QTL trait1	QTL trait2	QTL trait3
1	84,000,000	84,200,000	SNP1681 - SNP1685	x		x
1	14,500,000	14,750,000	SNP291 - SNP295		x	
2	92,500,000	92,450,000	SNP3847 - SNP3851	x		
2	46,700,000	46,900,000	SNP2935 - SNP2939			x
2	76,900,000	77,100,000	SNP3539 - SNP3543		x	
3	400,000	600,000	SNP4009 - SNP4013		x	
3	26,600,000	26,800,000	SNP4533 - SNP4537	x		
3	36,850,000	37,050,000	SNP4738 - SNP4742			x
4	7,650,000	7,850,000	SNP6154 - SNP6158			x
4	24,850,000	25,250,000	SNP6498 - SNP6502	x	x	
5	69,300,000	69,500,000	SNP9387 - SNP9391	x	x	
5	2,700,000	2,950,000	SNP8055 - SNP8059			x

### Estimation of the QTL effect for the whole window of 5 SNP

The QTL effect was calculated on the whole recorded population as follows.

For each sliding window, the most probable haplotype alleles were calculated using the EM algorithm [14], through Proc Haplotype in SAS [13], and were assigned to each phenotyped individual (n = 3000).

For each haplotype allele showing allele frequency ≥ .07 in the recorded population, the allelic substitution effect was estimated as a covariate on each trait, as in Sherman et al. [15], with the following model:

y = b(haplotype allele) + e

Where y = trait1, trait2 and trait3

Alleles were coded as follows: 2 copies of the same allele = 2; one copy = 1; no copy = 0.

To account for multiple testing, the corrected probability of the effect was estimated using the False Discovery Rate test with Proc Multtest in SAS [13].

### Estimation of the SNP effect from the haplotype effect

Under the hypothesis that one SNP of each haplotype was expected to have a major effect on the recorded trait, direct observation of those haplotype alleles that showed a highly significant effect (P < .00001) on one trait allowed to select one SNP where the two alleles showed opposite effects on that trait. For each of those SNPs, the substitution allelic effect was estimated as a covariate on each trait, similarly and with the same model as for the estimation of the allele haplotype effect.

## Results

Because the selective genotyping strategy was performed separately for the three traits, the statistically significant windows varied depending on the considered trait (Table 2).

The average additive relationship values of each of the selected tails, for each trait, were very similar to each other's (Table 3), ranging from 4.26 to 4.37 %; but they were higher than the corresponding value calculated for the whole population (3.01%). For all tested haplotypes, the corrected probabilities, after consideration of the FDR, of the allelic substitution effects, were reported in table 4.

**Table 3:** Average relationships in the selected groups of animals and in the whole population

Trait	tail	N	Coefficient of relationship	
			**mean**	**st dev**

1	highest	150	4.33	0.017
1	lowest	173	4.27	0.018
2	highest	167	4.32	0.017
2	lowest	165	4.27	0.018
3	highest	153	4.37	0.017
3	lowest	156	4.26	0.017
Total		4100	3.01	0.016

**Table 4:** Haplotype effects.

chr	Pos. (Mb) Start/End	**Haplo**. allele	Freq	Trait 1		Trait 2		Trait 3	
				**Effect**	**FDR *P***	**Effect**	**FDR *P***	**Effect**	**FDR *P***

1	84.0 84.2	11222	0.21	-48.7	<10^-4^	-0.5	ns	9.9*10^-3^	<10^-4^
		12121	0.24	32.5	<10^-4^	0.9	2*10^-2^	-4.1*10^-3^	<10^-4^
		12111	0.07	21.8	2*10^-2^	-0.2	ns	-7.0*10^-3^	<10^-4^
		11121	0.09	2.0	ns	0.3	ns	6.1*10^-4^	ns
		12122	0.07	24.2	1*10^-2^	-0.8	ns	-9.8*10^-3^	<10^-4^
		21222	0.07	-5.7	ns	0.1	ns	1.5*10^-3^	ns

1	14.5 14.7	11111	0.08	-16.7	7*10^-2^	-0.4	ns	2.1*10^-3^	8*10^-2^
		12111	0.19	37.1	<10^-4^	2.8	<10^-4^	2.9*10-3	5*10^-4^
		12112	0.18	18.1	4*10^-3^	0.5	ns	-2.2*10^-3^	8*10^-3^
		12121	0.14	0.5	ns	1.3	3*10^-4^	6.0*10^-3^	<10^-4^
		12212	0.32	-25.5	<10^-4^	-2.3	<10^-4^	-4.0*10^-3^	<10^-4^

2	46.7 46.9	11122	0.11	-14.1	ns	-0.5	ns	1.7*10^-3^	ns
		11112	0.26	-3.6	ns	0.4	ns	3.0*10^-3^	<10^-4^
		12111	0.23	-17.9	4*10^-3^	-1.0	2*10^-3^	4.3*10^-4^	ns
		22111	0.19	8.4	ns	0.0	ns	-2.4*10^-3^	6*10^-3^

2	76.9 77.1	11112	0.29	-12.9	2*10^-2^	-0.8	8*10^-3^	2.7*10^-6^	ns
		21112	0.28	2.4	ns	-0.1	ns	-1.2*10^-3^	ns
		22221	0.26	7.0	ns	0.8	1*10^-2^	1.3*10^-3^	ns
		22112	0.07	25.0	1*10^-2^	0.2	ns	-5.1*10^-3^	2*10^-4^

2	92.3 92.5	11111	0.39	13.1	2.*10^-2^	0.6	5*10^-2^	-5.1*10^-4^	ns
		22122	0.11	0.5	ns	0.1	ns	6.7*10^-4^	ns
		22222	0.11	-16.5	4*10^-2^	-0.6	ns	1.6*10^-3^	ns

3	0.4 0.6	11121	0.11	-22.1	8*10^-3^	-1.5	1*10^-4^	-1.7*10^-5^	ns
		11122	0.27	10.1	ns	-0.1	ns	-3.2*10^-3^	<10^-4^
		22211	0.32	9.5	ns	1.4	<10^-4^	3.5*10^-3^	<10^-4^
		22121	0.1	-9.0	ns	0.2	ns	3.4*10^-3^	3*10^-3^

3	26.6 26.8	11121	0.38	-17.3	4*10^-4^	-0.9	4*10^-4^	7.4*10^-4^	ns
		22212	0.22	31.7	<10^-4^	1.2	2*10^-4^	-3.3*10^-3^	<10^-4^
		11222	0.07	-15.9	ns	-0.9	ns	-6.2*10^-4^	ns
		22222	0.11	-4.8	ns	0.2	ns	2.4*10^-3^	ns

3	36.9 37.1	11111	0.14	24.4	1*10^-3^	-0.4	ns	-1.0*10^-2^	<10^-4^
		21111	0.11	-19.4	2*10^-2^	0.4	ns	7.4*10^-3^	<10^-4^
		21122	0.1	0.6	ns	0.6	ns	3.2*10^-3^	3*10^-3^
		21112	0.08	-2.3	ns	-0.2	ns	7.5*10^-4^	ns
		12111	0.16	-4.0	ns	-0.3	ns	-1.1*10^-1^	ns
		21222	0.19	-2.7	ns	0.4	ns	3.6*10^-3^	<10^-4^

4	7.7 7.9	11112	0.34	13.8	1*10^-2^	0.2	ns	-2.3*10^-3^	8*10^-4^
		11122	0.11	-14.9	5*10^-^2	-1.1	1*10^-2^	-6.4*10^-4^	ns
		12112	0.07	-25.8	1*10^-2^	-1.9	2*10^-4^	-1.8*10^-3^	ns
		22212	0.16	-13.9	4*10^-2^	0.2	ns	4.7*10^-3^	<10^-4^

4	24.9 25.3	11122	0.07	-12.3	ns	-1.8	1*10^-4^	-3.8*10^-3^	ns
		11211	0.15	-24.5	1*10^-4^	-1.3	1*10^-4^	8.2*10^-4^	ns
		12121	0.07	49.8	<10^-4^	3.0	<10^-4^	-6.0*10^-4^	ns
		12122	0.23	35.8	<10^-4^	2.6	<10^-4^	1.5*10^-3^	ns
		21111	0.14	-37.9	<10^-4^	-2.5	<10^-4^	-2.2*10^-5^	ns

5	69.3 69.5	12121	0.35	-27.7	<10^-4^	-1.6	<10^-4^	5.2*10^-4^	ns
		12112	0.15	20.2	2*10^-3^	0.8	4*10^-2^	-2.5*10^-3^	1*10^-2^
		12212	0.08	12.0	ns	-0.1	ns	-2.5*10^-3^	1*10^-2^
		21212	0.19	15.3	1*10^-2^	1.1	2*10^-3^	8.6*10^-4^	ns

5	2.7 2.9	12111	0.19	-11.4	ns	-0.4	ns	1.3*10^-3^	ns
		21221	0.16	-27.3	<10^-4^	-0.4	ns	4.9*10^-3^	<10^-4^
		22111	0.19	26.3	<10^-4^	0.8	5*10^-2^	-3.0*10^-3^	1*10^-4^
		22112	0.16	5.3	ns	-0.3	ns	-3.7*10^-3^	<10^-4^

Through direct observation of those haplotype alleles that showed a significant effect on one trait, it was possible to make evident which SNP, within the haplotype allele, might have been directly responsible of the trait variability. In Table 5 only the SNPs that presented a highly significant (P < .0001) allelic substitution effect were reported. These SNPs, located on chromosomes 1, 3 and 4 might be themselves considered the QTLs influencing the relevant trait.

**Table 5:** Effect of allele 1 of the SNP with major effect on each trait.

Chr	SNP	position	affected trait	effect	P-value
1	SNP293	14,600,000	2	2.470	2.0*10^-10^
1	SNP293	14,600,000	2	2.470	<1.0*10^-16^
1	SNP293	14,600,000	3	0.004	7.3*10^-09^
1	SNP1682	84,050,000	1	-39.230	<1.0*10^-16^
1	SNP1682	84,050,000	3	0.008	<1.0*10^-16^
3	SNP4738	36,850,000	3	-0.008	<1.0*10^-16^
4	SNP6155	7,700,000	1	18.050	8.0*10^-05^
4	SNP6155	7,700,000	3	-0.003	4.0*10^-05^
4	SNP6499	24,900,000	1	-58.550	<1.0*10^-16^
4	SNP6499	24,900,000	2	-3.760	<1.0*10^-16^

## Discussion

In this study, two assumptions were arbitrarily made. The first was that the selective genotyping strategy was successful for QTL mapping. Although the literature reported evidence of the suitability of this strategy [9], the decision to what animals should be considered as highly divergent for each trait was a choice of the authors. Therefore, the results obtained, both in numbers and in the position of the QTLs, might have been different if more or less restrictive parameters had been chosen. The additive relationship values of each of the selected tails, for each trait, were very similar to each other's, ranging from 4.26 to 4.37 %; but they were higher than the corresponding value calculated for the whole population (3.01%). To appraise the extent of the difference in the average relationship between the tails and the whole population, it is useful to cite Vahlsten et al. [16] who reported that an increase by 0.96 % units of relationship, per generation, is to be considered slow, this value referring to Friesian bulls, born during 40 years, and belonging to a population of over 400,000 animals. It can therefore be inferred that the relationship differences observed in the present study reproduce the mere generational trend.

The second assumption was that the QTL was encoded by an haplotype of 5 consecutive SNPs. Weller and Ron [17] underlined how important is the extent of LD in the application of genome scans to breeding programs. These authors noted that population-wide linkage LD extends, in dairy cattle, over less than 1 cM, i.e. a much shorter extent than the genetic linkage within families, that extends over tens of centimorgans. It is therefore possible that the hypothesis that the QTL was encoded by the haplotype with the highest effect on each trait was not the most appropriate for this study, the analyzed population consisting in a simulated sample. However, because the sliding windows encompass consecutive markers, the choice to select the top ranking window for each trait seemed appropriate, because it allowed the identification of single SNPs (Table 5) having a very high significant effect on one trait, the probability for some of them being < .1.0E-16.

## Conclusions

In this study we proposed a simple method to identify genomic regions associated to the phenotype under study, regions that could therefore be taken into account as the potential QTLs. The identification of the DNA region is the first step to identify the mutation which is really responsible for the variability of the trait, through the direct sequencing of the genomic regions that encode the QTL. The precision of the QTL estimation can vary depending on the deviations values established in the reference population to define which animals are extremely divergent.

## Competing interests

The authors declare that they have no competing interests.

## Authors' contributions

BM planned the study and applied the procedures to set up the sliding windows to be used in the subsequent analyses. GC and FN performed the statistical analysis of association providing the estimation of the QTL effects. All authors have contributed to the editing of the article, and approved the final manuscript.

## Declarations

This study is part of the GENZOOT research program, funded by the Italian Ministry of Agriculture (Rome, Italy).

This article has been published as part of *BMC Proceedings *Volume 8 Supplement 5, 2014: Proceedings of the 16th European Workshop on QTL Mapping and Marker Assisted Selection (QTL-MAS). The full contents of the supplement are available online at http://www.biomedcentral.com/bmcproc/supplements/8/S5
